# Case report of an intussusception presenting after a trauma jejunorrhaphy

**DOI:** 10.1016/j.amsu.2020.08.008

**Published:** 2020-08-14

**Authors:** Paul K. Lee, Antonio Masi, Ethan A. Warshowsky, Valery Roudnitsky

**Affiliations:** a Kings County Hospital, 451Clarkson Avenue, Brooklyn, N.Y, 11203, USA; b Downstate Medical Center, 450 Clarkson Ave, Brooklyn, NY 11203, USA

**Keywords:** Case report, Intussusception, Trauma, Suture lead point, Small intestine, Jejunorrhaphy

## Abstract

**Introduction:**

Intussusception in pediatric cases are predominantly idiopathic, while intussusception in adult cases are predominantly associated with a lesion. The differential diagnosis for the lesion includes Meckel's diverticulum, lipoma, adenoma, and metastatic disease.

**Presentation of case:**

We report a case of intussusception in which the lead point was the site of a jejunorrhaphy for a jejunal perforation secondary to blunt abdominal trauma. The intussusception presented as a postoperative bowel obstruction requiring a re-laparotomy and a segmental bowel resection. The postoperative course after the re-laparotomy was unremarkable.

**Discussion:**

Postoperative intussusception with a bowel anastomosis acting as the lead point is a rare but described complication of anastomotic procedures. Our report is the first in the trauma literature to describe an intussusception led by a jejunorrhaphy rather than a circumferential suture or stapled anastomosis. While rare, this complication is a critical constituent in the differential diagnosis of bowel obstruction after laparotomy for trauma. Currently, no standardized technique or prophylactic maneuver exists to prevent intussusception after an intestinal repair.

## Highlights

• Intestinal intussusception can occur in the immediate postoperative period of trauma jejunorrhaphy.
• Intestinal intussusception can occur in small, non-anastomotic intestinal repair.
• Prophylactic intestinal repair technique against intussusception is not yet established.

## 1 Introduction

Intussusception is a pathologic telescoping of a bowel segment through an adjoining segment. While the majority of pediatric cases are idiopathic, intussusception in adults is associated with a pathological lesion acting as a lead point in up to 93% of cases [1]. Such lesions may be benign (e.g. Meckel's diverticulum, lipoma or adenoma) or malignant (e.g. carcinoma or metastatic disease). Herein we report an atypical case in which the lead point was a jejunorrhaphy (lateral suture repair) of a traumatic jejunal perforation, in a community hospital. Of note, this report is in line with SCARE criteria [2].

### 1.1 Presentation of case

Our patient was a restrained passenger in a motor vehicle collision with airbag deployment. She was brought in by the emergency medical service to the trauma resuscitation area of the Emergency Department stable, complaining of severe abdominal pain with a diffusely tender abdomen. The patient was otherwise healthy and had no past medical, surgical, or family history. She additionally denied tobacco, alcohol or recreation drug use or any past psychosocial history. A computed tomography (CT) scan revealed air and free fluid in the peritoneal cavity. She was immediately taken for explorative laparotomy by the attending trauma surgeon and chief resident who discovered a single, one-centimeter perforation of the mid-jejunum on the antimesenteric side with the defect comprising <50% of the bowel circumference, indicative of a grade II small bowel injury by the American Association for the Surgery of Trauma [3]. The edges of the enterotomy were viable and well-perfused and thus no addition debridement was performed. The perforation was repaired primarily in a transverse orientation using a running 3–0 polypropylene suture with separate closures of the mucosal and seromuscular layers. There was minimal spillage of bowel content into the peritoneal cavity. No other injury was noted and the abdomen was closed.

**Fig. 1:**
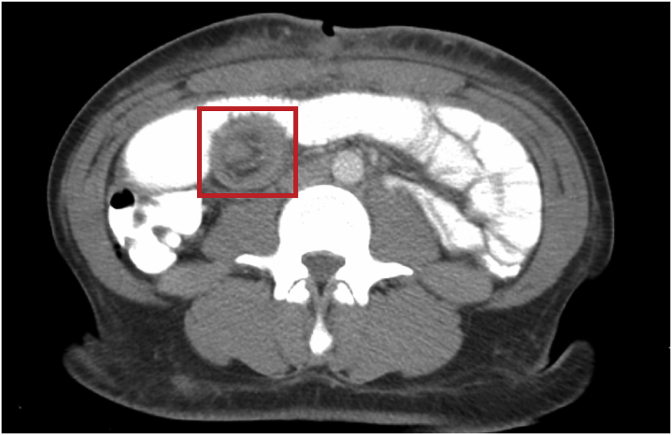
CT scan of the abdomen demonstrating an enteroenteric intussusception in the right-mid abdomen (at the center of the red box). (For interpretation of the references to colour in this figure legend, the reader is referred to the Web version of this article.)

Postoperatively, the patient failed to regain normal bowel function. The patient was also noted to have a distended abdomen, leading to a differential consideration of postoperative ileus, intraabdominal infection, and small bowel obstruction. A CT scan on the fifth postoperative day revealed an entero-enteric intussusception in the mid-abdomen causing a partial small bowel obstruction (Fig. 1). The patient was immediately taken to the operating room for a re-laparotomy, revealing an antegrade intussusception in the mid-jejunum with dilated loops of small bowel proximally. A gentle manual reduction of the intussusception showed that the transverse jejunorrhaphy was indeed the lead point. The repair site and the intussuscepted segment were both viable with neither leak nor hematoma. The involved segment was resected and remaining ends were anastomosed in a stapled side-to-side (functional end-to-end) fashion. The abdomen was closed after no other abnormality was noted (the trauma attending and chief resident performed the entire case). The patient made an uneventful recovery with return of gastrointestinal function on the second postoperative day. A 1-year follow up was unremarkable with no reported symptom or complication. The patient was grateful that she had endured her hospital stay without any long-term functional deficits from her trauma.

## 2 Discussion

Intussusception in which an anastomotic suture or staple line is the lead point is well-reported. It is especially common in patients with a Roux-en-Y gastric bypass, with intussusception being led by the gastro-jejunal anastomosis, or less frequently, by the jejunojejunal anastomosis [4]. Several cases of small bowel intussusception with the lead point at an end-to-end intestinal anastomosis have also been described [[5], [6], [7], [8], [9]]. In these cases the lead point was invariably a circumferential anastomosis.

Only one report described a repaired enterotomy as the lead point of a postoperative intussusception following a removal of an ileal phytobezoar [10]. The phytobezoar was removed though a longitudinal enterotomy and subsequently hand-sewn in a transverse orientation. The patient returned after a week with an anterograde intussusception with the suture repair as a lead point. Our case is the first description of this complication in the trauma literature.

While the precise mechanism of intussusception following a jejunorrhaphy of intestinal perforation and the prophylactic repair technique remain unestablished, the mechanism is likely in line with the mechanism of a local inhomogeneity in bowel wall as described by Reymond [11]. According to his proposed mechanism, a suture repair site may act as the local inhomogeneity in the bowel wall. A state of unstable equilibrium is created when the wall inhomogeneity is acted upon by peristalsis, later collapsing into a stable equilibrium observed as an intussusception. This process may further validate the value of excision of the involved segment and anastomosis in adult intussusception, as a lead point that has demonstrated the capacity to induce intussusception may do so again.

In cases of adult intussusception, excision of the involved segment is done so that the segment may undergo pathologic evaluation for malignancy given the lead point can be malignant in up to one half of cases [12]. However, for cases in which adult intussusception is known to be caused by a suture, further research is warranted to elucidate if the risk of recurrent intussusception due to the same lead point is less than the risk of anastomotic leakage and/or stenosis. Additionally, the morbidity of these two possible outcomes must be weighed. Furthermore, as pointed out above, the creation of a circumferential anastomosis poses it own risk of intussusception. There thus exists opportunity for further research supporting surgical management decisions in the case of adult intussusception caused by suture lead point.

## 3 Conclusion

The possibility of postoperative intussusception should be incorporated in the differential diagnosis of mechanical small bowel obstruction after a trauma laparotomy. As discussed above, intussusception may occur not only in patients after an intestinal anastomosis but also in those after a repair of only a portion of the intestinal wall circumference. Studies investigating the optimal technique for bowel repair and management of intussusception may further advance patient care and surgical outcome.

## Ethical approval

The study is exempt from ethical approval and the patient has given consent for the case report.

## Sources of funding

The authors have no funding source for the research.

## Author contribution

Paul Kyung Hyun Lee: validation, investigation, resources, writing – original draft, writing – review & editing, visualization, supervision, project administration. Antonio Masi: conceptualization, supervision, writing – review & editing. Ethan Avner Warshowsky: investigation, writing – review & editing, resource. Valery Roudnitsky: supervision, project administration, conceptualization

## Registration of research studies

The case report is not a first-in-man study.

## Guarantor

Paul Kyung Hyun Lee.

## Declaration of competing interest

None.
